# What spelling errors can tell us about the development of processes involved in children’s spelling

**DOI:** 10.3389/fpsyg.2023.1178427

**Published:** 2023-05-11

**Authors:** Georgia Z. Niolaki, Alexandra Negoita, Janet I. Vousden, Aris R. Terzopoulos, Laura Taylor, Jackie Masterson

**Affiliations:** ^1^School of Education, Bath Spa University, Bath, United Kingdom; ^2^School of Psychological, Social and Behavioral Sciences, Coventry University, Coventry, United Kingdom; ^3^Department of Psychology, Nottingham Trent University, Nottingham, United Kingdom; ^4^School of Psychology, Birmingham City University, Birmingham, United Kingdom; ^5^School of Psychology, Northampton University, Northampton, United Kingdom; ^6^Department of Psychology, UCL, Institute of Education, London, United Kingdom

**Keywords:** spelling, phonological plausibility, letter distance, automated measures of phonemes representations, primary age students

## Abstract

**Introduction:**

Spelling is an essential foundation for reading and writing. However, many children leave school with spelling difficulties. By understanding the processes children use when they spell, we can intervene with appropriate instruction tailored to their needs.

**Methods:**

Our study aimed to identify key processes (lexical-semantic and phonological) by using a spelling assessment that distinguishes different printed letter strings/word types (regular and irregular words, and pseudowords). Misspellings in the test from 641 pupils in Reception Year to Year 6 were scored using alternatives to binary correct versus incorrect scoring systems. The measures looked at phonological plausibility, phoneme representations and letter distance. These have been used successfully in the past but not with a spelling test that distinguishes irregularly spelled words from regular words and pseudowords.

**Results:**

The findings suggest that children in primary school rely on both lexical-semantic and phonological processes to spell all types of letter string, but this varies depending on the level of spelling experience (younger Foundation/Key stage 1 and older Key stage 2). Although children in younger year groups seemed to rely more on phonics, based on the strongest correlation coefficients for all word types, with further spelling experience, lexical processes seemed to be more evident, depending on the type of word examined.

**Discussion:**

The findings have implications for the way we teach and assess spelling and could prove to be valuable for educators.

## Introduction

Learning to spell is a lengthy and challenging process, and it is not surprising that some children struggle and need extra support (Nag et al., 2018). Therefore, it is important to find accurate and sensitive methods for assessing children’s spellings (Treiman and Kessler, 2004). One way to achieve this is to move away from the traditional binary correct vs. incorrect scoring method. Research has suggested that looking at children’s misspellings beyond the dichotomous scoring system might reveal patterns of development in linguistic knowledge (Masterson and Apel, 2010; de Bree and van den Boer, 2019; Treiman et al., 2019; for non-English orthographies see also Niolaki and Masterson, 2012; Carvalhais et al., 2020, 2021) and underlying cognitive processes that children use when they spell (Caravolas et al., 2001; Kessler, 2009; Treiman et al., 2016). These processes involve perception, attention, memory and expertise, all of which past research has explored in considerable depth (Georgiou et al., 2012; Rønneberg and Torrance, 2019; Zoccolotti et al., 2020; Niolaki et al., 2022). As such, to date, spelling has been primarily studied more in relation to cognitive abilities than to the actual spelling errors made. Recent research has suggested that it is worth looking in more depth at the characteristics of spelling attempts with different types of words (i.e., regular and irregular words and pseudowords; de Bree and van den Boer, 2019; Niolaki et al., 2020).

Dual route (DR) models of spelling have been proposed to explain how information is processed when spelling words (Barry, 1994). Similarly, models such as the integration of multiple patterns (IMP) model that support statistical learning (which is considered a powerful strategy for generalizing learning from untaught items) agree that both sublexical and lexical/semantic processes exist. The IMP model also recognizes that items can be divided into regularly and irregularly spelled words (Treiman, 2018, p. 648). These models suggest that people use sound-to-spelling rules, or *sublexical processing*, as well as stored knowledge of whole-word spellings, or *lexical processing* (Barry, 1994; Martin and Barry, 2012). Children are believed to possess the basis for lexical processing before they start to spell, as a result of spoken language and early exposure to printed text (Barry, 1994). Children are also believed to memorize the orthographic rules and learn to spell correctly as they gain more experience with print. As a result, children perform better on words that occur frequently in books (Kessler et al., 2012). A more pronounced frequency effect has been observed for irregularly spelled words (such as *DEBT*, *YACHT*, and *MORTGAGE*), indicating that children need to hear, read and write these words more times than regularly spelled words (such as *BARGE*, *SLATE*, and *TARGET*) in order to create a correct representation in memory (Kroese et al., 2000).

Empirical evidence suggests that novice spellers rely heavily or even exclusively on the sub-lexical route (Caravolas et al., 2001), and they progressively develop the lexical-semantic route. In the UK, phonics training is the starting fuel for children to understand how phoneme-grapheme correspondences operate. This is important as phonemes in English can have several spelling options (Barry and Seymour, 1988), for example, /k/ can be spelled <c>, <k>, or < ck>. The options vary in probability of occurrence, and this depends in part on whether context-free or context-sensitive probabilities are considered (context sensitive as in <magic—>magician>; Spencer, 2009). Overall, spelling encompasses different tiers of linguistic awareness (phonology, orthography, morphology and semantics), so to establish solid lexical representations, the spellers need to be aware of all these and their interrelationships.

To gain insight into the processes children use in spelling, sensitive, non-binary scoring systems have been developed to examine spelling errors (e.g., Bruck and Waters, 1988; Treiman and Kessler, 2004; Kessler, 2009). These systems have also been found to have good discriminatory power for students struggling with spelling (Treiman et al., 2016; for non-English languages see Niolaki et al., 2014; Joye et al., 2020). The non-binary measures rely, for example, on how close the misspelled word is to the correct spelling and can be used as a fine-grained measure to monitor the progress a child is making in developing spelling skills (Masterson and Apel, 2010). Many children, especially in the Foundation Year, produce more errors than correct responses (Georgiou et al., 2020; Carvalhais et al., 2021), and the measures can provide us with a clear picture of the use of lexical-semantic and sublexical processes, and how children’s reliance on these processes changes as they progress in spelling skill. Also, error analysis is not affected by floor effects which is an issue when we solely look at accuracy especially for young spellers (Treiman et al., 2019). We next present the measures we included in the current investigation.

Phonologically plausible errors (e.g., *elephant* spelled <elefant>) are considered to reflect use of sublexical spelling processes and are particularly apparent in novice spellers. Caravolas et al. (2001) analysed spellings from 153 children in the three first years in school in the UK. They emphasized the critical predictive role of phonological spelling ability for later reading and spelling accuracy. The researchers argued that children need phonics training (structured instruction that helps children to spell unfamiliar words) to build a solid sound-letter mapping system and then to become skilled spellers who competently use orthographic rules (Caravolas et al., 2001).

In a longitudinal and cohort study with 95 Portuguese students from two age groups—Grades 4–7 and 6–9, Carvalhais et al. (2021) conducted spelling error analyses and looked at phonological plausibility as one of the critical variables. They found that younger children made more misspellings than the older children and phonologically inappropriate errors were less in the older group. These findings are consistent with similar observations made in the English orthography (Treiman and Bourassa, 2000; Caravolas et al., 2001). The research highlights the importance of phoneme-grapheme associations in the earliest stages of spelling in English.

In the current study we employed two separate measures of phonological plausibility—a binary phonological plausibility score (PhP, e.g., spelling *elephant* as <elefant>), and an automatized continuous measure, the Automated Measure of Phoneme Representation (AMPR). The main difference between PhP and AMPR is the first is a binary measure (phonologically plausible error or not) and is hand scored (by the research team), whereas the second provides a score computed across the word. For AMPR, values closer to zero indicate a lower quality of error, as the target is distant from the response (Treiman and Kessler, 2004), meaning that as children’s spelling skill develops the AMPR score should increase. We also used letter distance (LD)^1^ in our analyses to capture the number of letter additions, deletions and substitutions needed to create the correct phonological and orthographic spelling from an error.

Treiman et al. (2016) investigated a range of scoring measures in a longitudinal study with children from Kindergarten to Grade 2. The findings indicated that children possess some phonological knowledge early in spelling development, however, LD, the lexically-related measure, proved a better predictor of spelling accuracy in beginner spellers than the phoneme distance measure, the sublexically-related one (Treiman et al., 2016). We considered that it would be informative to see, in our study with UK children, and almost 21 years after the implementation of synthetic phonics in schools, whether PhP or LD would be more strongly associated with emergent spelling, and whether the association would differ for regular words, irregular words and pseudowords, as these are assumed to draw on different processes, that is, whole-word and sublexical. For LD, values that are closer to zero indicate less distance from the correct spelling. Thus, as children develop their spelling knowledge LD values should decrease.

Several studies have reported that more sensitive scoring methods can effectively capture developmental changes in spelling and strong associations with reading and phonological ability (Ritchey et al., 2009; Clemens et al., 2014; Frisby, 2016). As noted above, Treiman et al. (2016) investigated a range of measures in a longitudinal study. Participants were 374 children from kindergarten age to Grade 2 in the USA and Australia. The researchers employed letter-based measures (LD, correctness, letter sequence) and phoneme-based measures (AMPR, phoneme distance, sound-spelling) at two-time points. The findings revealed that letter-based measures accounted for more variance in spelling accuracy than phoneme-based measures. Treiman et al. (2019) reported a replication study with British English spellers where correctness (a binary correct/incorrect measure) was more predictive of single word spelling at Time 4 assessment (when the children attended the spring term of Year 2) than non-binary measures. Binary measures might be useful for predicting who is likely to struggle with spelling but would not tell us why. In this case non-binary measures would give us more insight.

Carvalhais et al. (2020) in their study with Portuguese students (also reported earlier) found that orthographically related variables (such as stress mark errors and orthographic misspellings) were the most common errors in older learners (Grades 4, 6, and 7). In the English-based studies reported above children were tested in the earlier school grades, so there is not much evidence for English on how the phonological- and orthographic-related measures perform in older spellers.

In summary, it has not been established if developmental changes in spelling errors apply equally to regular and irregular words and pseudowords. Moreover, several scoring methods have been found to relate to one another, for example, PhP and AMPR (Treiman et al., 2019), yet they have not been investigated concerning spelling errors with different letter string/item types. The current study addressed these gaps in the literature by exploring patterns of spelling errors in primary school children split into three age groups, Foundation Year/Key Stage 1 (F/KS1; Kindergarten to grade 2), Early Key Stage 2 (EKS2; Grade 3 to 4) and Advanced Key Stage 2 (AKS2; Grade 5–6). Thus, the goal of the present study was to investigate the strategies children use when they spell different types of words, through analysis of their errors, and how this may change from reception year to year 6 (5- to 12-years).

## The current study

We aimed to investigate whether children rely on different processes for different types of words in their spelling, and explored whether this reliance might change from Reception year to Year 6 (equivalent to Kindergarten to Grade 6 in the USA). Research has indicated that both lexical-semantic and sub-lexical processes are employed for spelling by beginner spellers, while the former processes seem to become more prevalent when children gain more experience with reading and spelling (Bruck and Waters, 1988; Caravolas et al., 2001; Kessler et al., 2012; Share, 1995). Treiman et al. (2016, 2019) found that lexically related variables were strong predictors of more advanced spelling (however, the older children only went up to Grade/Year 2). When investigating the spelling of different types of letter strings, it is likely that children rely more on sub-lexical processes for pseudowords, and lexical processes for irregular words, while the spelling of regular words will tap both processes (Niolaki et al., 2022). Based on the research reviewed above, we aimed to test the following hypotheses. We expected that the sub-lexically related (phoneme-based) measure AMPR would be more important for spelling pseudowords than irregular words. We also anticipated that the sublexically- and lexically-related (letter based) measure LD would be important for real words; but less strongly associated with pseudoword spelling.

Specifically, we made the following predictions:

- There will be a significant interaction between key stages and word types for spelling accuracy, letter-based and phoneme-based measures. Lower key stages should evidence lower scores on accuracy and phoneme-based scores and higher scores on letter-based measures for each word type than higher key stages.
- There will be significant associations between phoneme-based and letter-based measures and accuracy for all word types in all key stages. The strength of associations will vary depending on key stage and letter string type.

## Methods

### Participants

Participants were 641 UK primary school children attending Reception Year to Year 6, from a mix of urban and rural schools (seven different state schools). School years were divided into three levels as follows: Group 1 comprised children in Foundation /Key Stage 1 (F/KS1), i.e., Reception Year to Year 2, Group 2 were children in the first half of Early Key Stage 2 (EKS2), i.e., Year 3 and 4, and Group 3 were children in the second half of Advanced Key Stage 2 (AKS2), i.e., Year 5 and 6. Pupils were grouped this way to allow for the identification of strategies used for spelling in developmental stages (beginning, early and late stages/advanced spellers). The number of children in each age group, together with their mean chronological age, is shown in Table 1. Data were collected after parents/carers of participating students returned consent forms and after children assented to participate in the study. The University Ethics’ Committee granted ethical approval for the study.

**Table 1:** Number and mean chronological age of participants per age group (standard deviations are in parentheses).

	F/KS1	EKS2	AKS2
Number of participants	309	165	167
Age (years)	6.34 (0.95)	8.77 (0.94)	10.78 (0.56)

### Materials

Data from a new interpretive spelling test for primary school children were collected. The test consists of three sections comprising 36 irregular words (e.g., <yacht>), 36 regular words (e.g., <cat>), and 34 pseudowords (e.g., <trelfishly>). Spelling regularity was calculated based on the frequency of occurrence of sound-letter correspondences in the word (Spencer, 2009; Vousden et al., 2011) but also spelling instruction in UK schools. Pseudowords were formed by combining the first half of a regular word and the last half of another regular word. When spelled, these items follow regular words’ structure, but they were unfamiliar to the children. For pseudoword accuracy (using binary scoring) we categorized any plausible spelling as accurate. The first and third authors agreed on the phonologically plausible acceptable responses for the 36 pseudowords (for example the item <clep> was phonologically plausible if spelled as <clepp> and <klep>).

Items were matched across the three subtests on word length, and across regular and irregular words on zipfrequency and zipf contextual diversity (see Niolaki et al., 2022,^2^ for details). The reliability of each subtest is high based on the accuracy scores of the binary assessment: irregular words *α* = 0.97, regular words *α* = 0.96, and pseudowords *α* = 0.94. Each spelling response was scored using binary, non-binary and categorical measures. Table 2 provides examples of scoring with the different measures.

**Table 2:** Examples of scoring using all scoring measures.

Target word	Child’s spelling	Accuracy	PhP	AMPR	LD
*Life*	*Live*	*0*	*0*	*0.66*	*1.4*
*Nature*	*Nocher*	*0*	*0*	*0.66*	*6.2*
*Flavor*	*Flaver*	*0*	*1*	*1*	*2.4*
*Aspire*	*Aspier*	*0*	*1*	*1*	*2*

PhP, phonological plausibility; AMPR, automated measure for phoneme representation; LD, letter distance.

### Accuracy

Children’s spellings were given a score of zero for incorrect and one for correct spellings. The maximum possible accuracy score was 106.

### Orthographic measure

#### Letter distance

Letter distance was calculated using Ponto [available online at http://spell.psychology.wustl.edu/ponto/ (Kessler, 2009)]. This online tool allocates points for each deletion, addition, transposition or substitution needed for the child’s written response to be transformed into the conventional spelling. Mean distance scores were generated for each child for regular words, irregular words and pseudowords separately.

### Phonological measures

#### Phonological plausibility

An error was given a score of one if it was phonologically plausible and zero if there were incorrect phoneme-grapheme correspondences (phonemic error), or if additional elements were present or absent. Errors were calculated as PhP or not by the second author and agreed by the first and third authors.

#### Automated measures for phoneme representation

The AMPR scoring metric comprises the number of phonologically plausible phonemes in a word divided by the total number of phonemes. The AMPR calculates a lower score when phonologically implausible errors are made, for example, *life* spelled as *live* would receive a score of 0.66 (2/3 phonemes correctly represented) while *life* spelled *life* would receive a score of 1 (3/3 phonemes correctly represented, the highest score). The measure was generated using the online software (available at http://spell.psychology.wustl.edu/AMPR), yielding scores between 0 and 1, where scores nearer 1 represent a phonologically plausible response and scores closer to zero represent a non-phonologically plausible response (Treiman and Kessler, 2004; see Table 3). Mean scores for each word type per child were calculated.

**Table 3:** Means for all scoring measures (standard deviations are in parentheses).

		Accuracy%	PhP%	AMPR	LD
Year_groups	Type of word				
	Irregular	17.1 (19.9)	26 (18)	0.78 (0.19)	3.28 (1.55)
F/KS1	Regular	38.5 (25.9)	16 (13.5)	0.77 (0.18)	1.86 (1.57)
	Pseudoword	34.4 (22.7)	-	0.72 (0.17)	2.12 (1.66)
	Irregular	54.8 (23.1)	42.3 (20.8)	0.89 (0.07)	1.31(0.88)
EKS2	Regular	74.2 (18.8)	23.7 (21)	0.86 (0.09)	0.57 (0.58)
	Pseudoword	62.7 (17.1)	-	0.86 (0.26)	0.83 (0.65)
	Irregular	76.9 (17.2)	61.8 (24.9)	0.95 (0.04)	0.54 (0.47)
AKS2	Regular	86.4 (11.4)	34.1 (29.7)	0.91 (0.06)	0.24 (0.22)
	Pseudoword	70.1 (12.8)	-	0.85 (0.07)	0.61 (0.37)

PhP, phonological plausibility; LD, letter distance; AMPR, automated measure for phoneme representation.

### Procedure

The data were collected in Spring/Summer term by researchers and trained research assistants testing children in small groups in urban and rural primary schools (at least three research assistants supervised the children, and the class teacher also helped during the procedure). For details of the procedure please see Niolaki et al. (2022). Error analysis was computer-scored or hand-scored, as described above, by the authors. In the case of scoring by hand, categorisations were discussed and agreed upon between the first, second and third authors. Agreement between authors had to reach 100% for hand-scored variables.

## Results

Total correct scores were computed for all participants for regular words, irregular words and pseudowords. Average scores for AMPR and LD were computed. Means and standard deviations per measure and word type are presented in Table 3.

**Figure 1:**
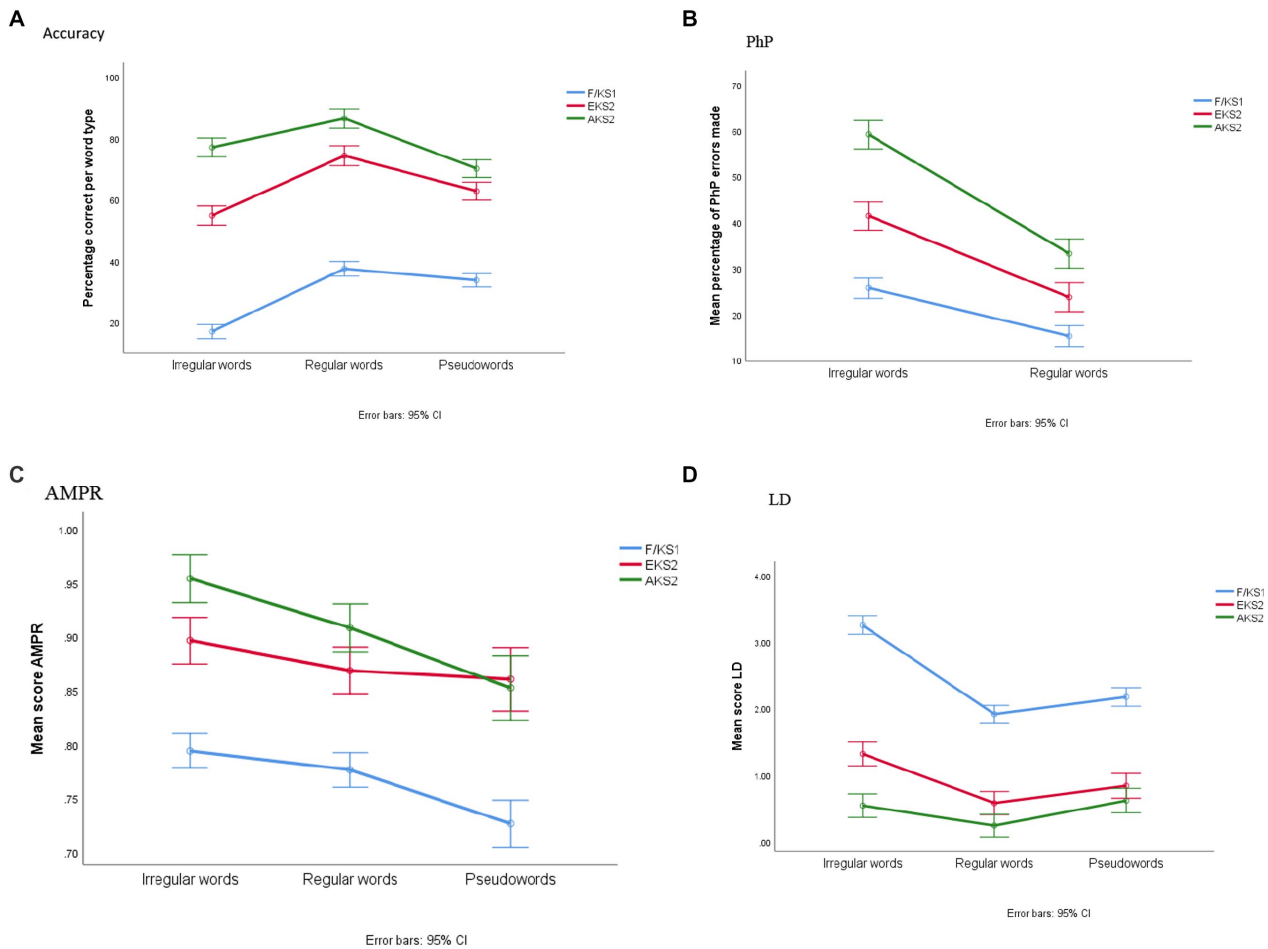
Mean accuracy, phonological plausibility (PhP), automated measure for phoneme representation (AMPR), and letter distance (LD) score for each word category and group.

A two-way Analysis of Variance (ANOVA) was conducted to explore Key stage group differences in accuracy. The between groups variable was Key stage group (F/KS1 (Reception Year to Year 2), EKS2 (Year 3 and Year 4), AKS2 (Year 5 and Year 6)), and the within groups variable was word type (regular word, irregular word, pseudoword). There was a significant interaction between word type and Key stage groups [*F*(4, 1,240) = 76.44, *p < 0*.001, *η*_p_^2^ = 0.198]. While pseudowords were spelled more accurately than irregular words in F/KS1 and EKS2, this reversed in AKS2 (see Figure 1A). Regular words were the most accurate in all key stages. There was a significant main effect of word type [*F*(2, 1,240) = 402.7, *p* < 0.001, *η*_p_^2^ = 0.394]—the children were more accurate in spelling regular words (*M* = 23.8) than irregular words (*M* = 19.9) and pseudowords (*M* = 17.9; *p* < 0.001). There was also a significant main effect of group [*F*(2, 620) = 423.6, *p* < 0.001, *η*_p_^2^ = 0.577], with AKS2 children spelling significantly more items accurately overall (*M* = 28.0) than EKS2 (*M* = 23.0) and F/KS1 (*M* = 10.6; all *p*_s_ < 0.001). The difference between EKS2 and F/KS1 was also significant.

The same analysis was carried out for the PhP, AMPR and LD scores (see Figures 1B–D for plots of the mean scores). For PhP the scores were for regular and irregular words only as all phonologically plausible responses for pseudowords were counted as correct. The results of the two-way ANOVA revealed a significant interaction of word type and Key stage group [*F*(2, 601) = 23.3, *p* < 0.001, *η*_p_^2^ = 0.07]. The main effect of word type was significant [*F*(1, 601) = 341.9, *p* < 0.001, *η*_p_^2^ = 0.36] as children made more PhP errors for irregular (*M* = 42.1) than regular words (*M* = 24.1). The main effect of group was significant [*F*(2, 601) = 125.8, *p* < 0.001, *η*_p_^2^ = 0.29]. The difference between F/KS1 (*M* = 46) and EKS2 (*M* = 23) and AKS2 (*M* = 11) was significant, as was that between EKS2 and AKS2.

For the AMPR scores, the interaction of word type and key stage group was significant [*F*(4, 1,162) = 5.37, *p* < 0.005, *η*_p_^2^ = 0.018]. AMPR scores increased from F/KS1 to AKS2 for each word type except pseudowords. In the case of pseudowords, scores increased from F/KS1 to EKS2 only. There was a significant main effect of word type [*F*(2, 1,162) = 64.8, *p* < 0.001, *η*_p_^2^ = 0.100] with significantly lower scores for pseudowords (*M* = 0.81) than regular words (*M* = 0.85) and irregular words (*M* = 0.88; *p*s < 0.01 for all differences). The difference between regular and irregular words was also significant (*p* < 0.001). There was also a main effect of group [*F*(2, 581) = 65.06, *p* < 0.001, *η*_p_^2^ = 0.183]. F/KS1 had significantly lower scores (*M* = 0.766) than ESK (*M* = 0.875) and ASK2 (*M* = 0.905) but the difference between ESK2 and ASK2 did not reach significance.

Finally, for LD, there was a significant interaction between word type and key stage group [*F*(4, 1,220) = 125.9, *p* < 0.001, *η*_p_^2^ = 0.29]. LD scores decreased from F/KS1 to EKS2 for each word type. Irregular words had the highest score but in AKS2 pseudowords scored higher. There was a significant main effect of word type [*F*(2, 1,220) = 525.7, *p* < 0.001, *η*_p_^2^ = 0.46]. There were significantly higher scores for irregular words (*M* = 1.71) than regular words (*M* = 0.91) and pseudowords (*M* = 1.21; all *p*s < 0.001). The difference was also significant between regular words and pseudowords (*p* < 0.001). There was a significant main effect of group [*F*(2, 610) = 197.4, *p* < 0.001, *η*_p_^2^ = 0.39]. F/KS1 had significantly higher scores (*M* = 2.45) than EKS2 (*M* = 0.91) and AKS2 (*M* = 0.47; all *p* < 0.001). The difference between EKS2 and AKS2 was also significant (*p* < 0.001).

### Correlation analyses

Partial correlations were conducted for each KS group, controlling for grade, in order to explore the associations of the phoneme and letter-based measures with spelling accuracy for regular words, irregular words and pseudowords for AMPR and LD, and for regular and irregular words for PhP. The results are presented in Table 4.

**Table 4:** Correlations between spelling accuracy and scoring metrics controlling for year_group.

		F/KS1 accuracy			EKS2 accuracy		AKS2 accuracy		
Scoring metric	Ir	Rg	Pw	Ir	Rg	Pw	Ir	Rg	*Pw*
PhP Ir	0.391***	-		0.435***	-		0.446***	-	
PhP Rg	-	0.420***		-	0.334***		-	0.202*	
									
AMPRIr	0.290***	-	-	0.456***	-	-	0.411***	-	-
AMPR Rg	-	0.529***	-	-	0.237**	-	-	0.187*	-
AMPRPw	-	-	0.606***	-	-	−0.073	-	-	0.170*
									
LD Ir	−0.719***	-	-	−0.951***	-	-	−0.956***	-	-
LDRg	-	−0.682***	-	-	−0.943***	-	-	−0.930***	-
LD Pw	-	-	−0.751***	-	-	−0.882***	-	-	−0.927***

*p < 0.05, **p < 0.01, ***p < 0.001. Ir, irregular; Rg, regular; Pw, pseudowords; PhP, phonological plausibility; AMPR, automated measure for phoneme representation; LD, letter distance.

For the F/KS1 group, significant associations were observed between accuracy for all word types and all measures. For EKS2, all associations were significant, with the exception that AMPR pseudoword scores were not associated with pseudoword accuracy. Notably, for AKS2, associations with PhP and AMPR for regular words are lower than for the youngest age group. High levels of accuracy could explain these weaker associations in the older age groups. However, LD was consistently strongly correlated with accuracy across the age groups and letter string types.

In order to determine differences in the associations Eid et al.’s (2011) comparison of correlations (online calculator^3^) was used. LD and AMPR were compared, as PhP was less strongly associated with accuracy according to the results of the correlations (see Table 4).

### Comparison of correlations for F/KS1

The relationship between irregular word accuracy and AMPR irregular word score (*r* = 0.290) was significantly less strong than that for regular word accuracy and AMPR regular word score (*r* = 0.529; *z* = 5.1, *p* < 0.001). The association between regular word accuracy and AMPR regular word score (*r* = 0.529) was significantly lower than that for pseudoword accuracy and AMPR pseudoword score (*r* = 0.606; *z* = 1.9, *p* < 0.05).

There were no significant differences in the associations between LD scores and accuracy for regular vs. irregular and pseudoword vs. irregular. Unexpectedly, the coefficient for LD regular words and regular word accuracy (*r* = −0.682) was significantly less strong than the coefficient for LD pseudowords and pseudoword accuracy (*r* = −0.751; *z* = 2.4, *p* < 0.01).

### Comparison of correlations for EKS2

The coefficient for irregular word accuracy and AMPR irregular words (*r* = 0.456) was significantly higher than that for regular word accuracy and AMPR regular words (*r* = 0.237; *z* = 3.2, *p* < 0.001). This is different to what we found for F/KS1 children. The difference between the coefficient for LD irregular words/irregular word accuracy and the coefficient for LD regular word/ regular word accuracy was not significant, indicating reliance on lexical processes for both word types. The coefficient of the association between LD and accuracy for pseudowords was significantly lower than for irregular words (*z* = 5.86, *p* < 0.001) and regular words (*z* = 4.87, *p* < 0.001).

### Comparison of correlations for AKS2

The difference between the coefficient for irregular word accuracy and AMPR irregular words (*r* = 0.411) and regular word accuracy and AMPR regular words (*r* = 0.187) was significant (*z* = 10.1, *p* < 0.001). This is consistent with the findings for EKS2 but not for F/KS and this is to be expected due to the consistent sound—letter associations that the regular words have.

The coefficient for LD irregular words and irregular word accuracy (*r* = −0.956) was significantly higher than that for LD regular words and regular word accuracy (*r* = −0.930), (*z* = 3.3, *p* < 0.001). This suggests that for irregular words there is more reliance on lexical processes than for regular words. The coefficient for LD irregular words and irregular word accuracy was also significantly higher than the coefficient for LD pseudowords and pseudoword accuracy (−0.927; *z* = 3.3, *p* < 0.001). This indicates that pseudowords in comparison to irregular words rely less on lexical processes.

## Discussion

This study aimed to examine the interactions between key stages, spelling accuracy and measures of sublexical (phonological plausibility: PhP, Automated Measures for Phoneme Representation: AMPR) and lexical spelling processes (Letter Distance: LD). Spelling was examined in relation to the type of word, regular and irregular words and pseudowords, as they are differentially affected by lexicality. Beginning spellers seem to rely more on sublexical processes for spelling, and as children gain experience, lexical processes become more important. It was expected that spelling of irregular words would be primarily associated with lexically-related variables, while spelling of pseudowords would be associated with sublexically-related variables. For regular words, there should reliance on both lexical and sublexical processing.

In terms of accuracy, similarly to past findings (de Bree and van den Boer, 2019), it was observed that children were the most accurate in spelling regular words, for all key stages. While pseudowords were spelled more accurately than irregular words up to the second half of KS2, the AKS2 children were more accurate with irregular words than pseudowords. This suggests that as they gain more experience with spelling and reading, children rely more on orthographic (lexical) processes. This is supported by the significant interaction between KS and LD, with improving scores for LD for all letter string types from early KS to AKS. The improvement for irregular words was such that they outperformed pseudowords at AKS2. The results of the present study are in line with the findings of former studies which indicated that spelling development is continuous rather than stage driven, reflecting gradual improvements in children’s phonological and orthographic knowledge (de Bree and van den Boer, 2019; McMurray, 2020; Carvalhais et al., 2021).

AMPR also significantly improved from early to advanced KS for regular and irregular words. Pseudowords improved between F/KS1 and EKS2 but remained similar between EKS2 and AKS2, highlighting that as children put more effort into applying lexical processes at AKS2, phonologically implausible errors decrease more for irregular and regular words than pseudowords. This implies that for real words, phonological plausibility becomes strongly linked to lexical processing and automatic retrieval, which is also indicated by stronger associations between AMPR and accuracy as KS advances, when for pseudowords these associations become weaker. Another marker of sublexical processing, PhP, confirmed the regular word superiority over irregular words, as irregular words had consistently more phonologically appropriate errors than regular words. However, this result does not capture the subtle changes and improvements as shown by the AMPR and KS interaction effects as AMPR can be used for pseudowords. The results endorsed past research suggesting that non-binary measures are good metrics with which to monitor spelling development (Masterson and Apel, 2010; Werfel and Krimm, 2015).

To further confirm this, the strength of the relationships between spelling accuracy and phoneme- and letter-based measures differed as a function of KS and the type of letter string. Across KS groups, the strongest associations were found with LD, and less so for PhP and AMPR. The discrepancy in the strength of associations between LD and phoneme-based measures might be also explained by the inclusion of multisyllabic words in the spelling test, that need more effort at the whole-word level (Heggie and Wade-Woolley, 2017) and the different types of letter stings that have differential reliance on lexical and sublexical processing. This may explain the difference to Treiman et al. (2016, 2019), who found that the strongest predictors of spelling were accuracy followed by PhP and then LD.

For the early F/KS1 spellers the association between sublexical processes (i.e., AMPR) and accuracy for regular words and pseudowords was stronger than that for irregular words. This is expected as irregular words have less predictable letter-sound associations. At EKS2 the association between AMPR pseudoword and pseudoword accuracy was non-significant and smaller in comparison to the coefficients for regular and irregular words. This may suggest that at this stage knowledge of how the word is spelled is more important than sublexical phoneme-grapheme knowledge, which, by this KS level reaches a plateau.

Similarly, at AKS2, the association of irregular word accuracy with AMPR was stronger than the association of regular word accuracy and AMPR. While the influence of phonics in Key Stage 2 becomes less pronounced, for irregular words sublexical processes can be still important due to the inconsistent phoneme-grapheme correspondences. At KS2 there is more reliance on lexical processes (reflected in LD scores) for irregular words than for pseudowords, which is consistent with the findings for F/KS1. It is also noteworthy that AMPR and PhP show consistently significant and fairly similar in strength associations for regular and irregular words in all KS. This finding confirms that the two variables tap the same construct.

At F/KS1 LD was more strongly associated with accuracy for pseudowords than regular words. This is likely because regular words benefit from being able to draw on both lexical and sublexical processes so will be the closest to the target spellings. In the case of pseudowords, they need to be spelled using phoneme-grapheme correspondences (PGCs) and we counted any legal alternative PGC as correct (unlike in the case of the words where it must be only the target one for that word in order to be correct). This lenient criterion for the pseudowords means they can be accurate phonologically while having many letters different from the target. In the case of the irregular words, the children will be trying to use PGCs but these will be disadvantageous and will lead to a big difference in letters compared to the target.

At EKS2 the difference in the associations between LD and accuracy for regular and irregular words did not reach significance indicating reliance on lexical processes for both word types, demonstrated also by the significantly stronger associations in comparison to the LD-accuracy association for pseudowords. However, at AKS2 LD-accuracy irregular word associations were stronger than those for both regular words and pseudowords, showing a shift to relying more on lexical processes when spelling irregular words.

AMPR and LD are two non-binary measures that relate to spelling accuracy with all letter string types (except AMPR and pseudoword accuracy at EKS2). The strength of associations varied depending on the group and letter string category. AMPR was consistently less strongly associated with accuracy in comparison to LD. For pseudoword and regular word accuracy this is not an odd finding as for children with more competence in spelling, sublexical processing will be less critical for their spelling due to the straightforward PGCs. For irregular word spelling, the reliance on lexical processes is strong at later stages, as reflected in the LD scores outperforming LD for pseudowords at AKS2. For FKS1 children there is also less influence of phoneme-based measures (AMPR) than LD for irregular word spelling even if the influence of phonics teaching is strong in this age group. The influence of phonics, although strong, is not the optimal strategy to spell irregular words. Regular words, on the other hand, at all KS, are the most accurate word type as they benefit from input from both lexical and sublexical processes, indicated also by the strong associations between LD scores and accuracy. The findings suggest that there is a gradual unification of spelling processes (orthography, phonology and semantics) from KS1 to AKS2 as suggested by the lexical quality hypothesis (Perfetti and Hart, 2002) and the ‘linguistic trilogy’ that suggests spelling effectively requires all three processes (Wolter and Dilworth, 2014).

Finally, for pseudoword spelling and for F/KS1, there was influence of LD and for EKS2, LD was more strongly associated with pseudoword accuracy than with regular and irregular word accuracy. This may be partly explained to the way pseudowords were devised for the spelling test, which implicates lexical processing. In an attempt to spell pseudowords that somewhat resemble real words, children employ lexical processes to a greater extent than for regular and irregular words, as the latter two are more easily recognized. Lexical processing becomes a strategy that can be transferred to other types of letter strings. Another potential interpretation could be that pseudowords need to be spelled using PGCs and we counted any alternative PGC as correct. This lenient criterion for the pseudowords means they can be accurate phonologically while having many letters different from the target.

This important finding agrees with results from the longitudinal study of Carvalhais et al. (2021). Those researchers found for the older cohort of Portuguese learners that orthographic and stress errors were more prevalent than phonological errors, indicating that the influence of lexical semantic processes are stronger as phonological skills are by now very well mastered. That means from a school perspective that spelling instruction should foster more orthographic strategies in these later stages of education.

Use of non-binary ways to score spelling errors can provide the teachers with a powerful tool to unpick the type of errors children make and if they need more support in phonological and lexical semantic (orthographic elements) processes. The spelling test with irregular and regular words and pseudowords can further uphold the teacher’s work in finding the linguistic gaps that children might have and tailor an appropriate bespoke intervention. It is also evident that in addition to phonological processes, orthographic knowledge plays a significant role in learning to spell. Therefore, an educational system that emphasizes solely alphabetic strategies may put children at a disadvantage and it might also be of disservice.

As with any study, this one is not free of limitations. Several variables were generated by computer programs; however, some errors can still be difficult to categorize. Also, the data are cross-sectional while a longitudinal design could be more informative. However, these limitations are minimized by strengths, such as the reliable measures that were used to score children’s spellings. PhP has been employed in many earlier studies examining the type of errors children make. The large sample size (N = 641) and items in the spelling test (N = 106) produced a large number of spellings—the total number of words including correct answers and no responses exceeded 65,000. To further our understanding of spelling development, researchers could apply a similar method in a cross-cultural study where spellings of native speakers of other orthographies are examined. This will allow us to ascertain whether these findings are universal or not.

Many years of schooling need to take place in order for spellings to become established entries in the mental lexicon. This study is one of the first to demonstrate when the lexical and sublexical processes based on the DR model (Martin and Barry, 2012) start to function in a more integrated and interdependent way. It will be informative for further studies to be conducted with more transparent and opaque orthographies as these can indicate if there is a universal age at which this occurs.

## Conclusion

The scoring measures included previously showed primary school children’s reliance on lexical and sublexical spelling processes (Caravolas et al., 2001; Treiman et al., 2016, 2019). The measures assign credit for partially correct spellings and can allow researchers and educators to capture changes in the development of spelling ability, and to analyse spelling performance (Treiman and Kessler, 2004; Kessler, 2009; Masterson and Apel, 2010). The fine-tuned measures employed in calculating the spelling errors, in addition to use of an assessment that differentiates between different word types, means we were able to capture changes as the children become more advanced in spelling. It was observed that as spelling skill increased, and the influence of systematic synthetic phonics presumably became less strong, children appeared to become more reliant on lexical processes. We believe that our study will inspire more research in spelling which will help unlock the mystery of learning to spell conventionally, and highlight that every letter counts for understanding spelling processes.

## Data availability statement

The raw data supporting the conclusions of this article will be made available by the authors, without undue reservation.

## Ethics statement

The studies involving human participants were reviewed and approved by Bath Spa University. Written informed consent to participate in this study was provided by the participants’ legal guardian/next of kin.

## Author contributions

All authors listed have made a substantial, direct, and intellectual contribution to the work and approved it for publication.

## Funding

This article was supported by Research England HEQR seed funding from Bath Spa University and British Academy (BA)/Leverhulme Small Research Grant [SG170586] awarded to the GN and a British Psychological Society Grant awarded to the AN.

## Conflict of interest

The authors declare that the research was conducted in the absence of any commercial or financial relationships that could be construed as a potential conflict of interest.

## Publisher’s note

All claims expressed in this article are solely those of the authors and do not necessarily represent those of their affiliated organizations, or those of the publisher, the editors and the reviewers. Any product that may be evaluated in this article, or claim that may be made by its manufacturer, is not guaranteed or endorsed by the publisher.

## Footnotes

^1^LD: calculated using Ponto http://spell.psychology.wustl.edu/ponto/ (Kessler, 2009).

^2^A list of the items is in the appendix of the published paper Niolaki et al. (2022).

^3^Eid et al. (2011): https://www.psychometrica.de/correlation.html#dependent.
